# Maternal perceptions of childhood vaccination: explanations of reasons for and against vaccination

**DOI:** 10.1186/s12889-018-6338-0

**Published:** 2019-01-10

**Authors:** Deborah A. McNeil, Melissa Mueller, Shannon MacDonald, Sheila McDonald, Vineet Saini, James D. Kellner, Suzanne Tough

**Affiliations:** 1 Maternal Newborn Child and Youth Strategic Clinical Network, Alberta Health Services, Calgary, Alberta Canada; 2 University of Calgary, Faculty of Nursing and Cumming School of Medicine Department of Community Health Sciences, Calgary, Alberta Canada; 3 Alberta Children’s Hospital Research Institute, Calgary, Alberta Canada; 4 Research and Innovation, Population Public and Indigenous Health, Alberta Health Services, Southport Atrium, 10101 Southport Road S.W, Calgary, Alberta T2W 3N2 Canada; 5 Faculty of Nursing, University of Alberta, Edmonton, Alberta Canada; 6 Cumming School of Medicine, Department of Pediatrics, University of Calgary, Calgary, Alberta Canada; 7 University of Calgary Faculty of Veterinary Medicine, Calgary, Alberta Canada; 8 Departments of Pediatrics and Community Health Sciences, University of Calgary Cumming School of Medicine, Calgary, Alberta Canada

**Keywords:** Childhood vaccination, Immunization, Maternal perspective, Qualitative analysis

## Abstract

**Background:**

Understanding reasons for and against vaccination from the parental perspective is critical for designing vaccination campaigns and informing other interventions to increase vaccination uptake in Canada. The objective of this study was to understand maternal vaccination decision making for children.

**Methods:**

Mothers participating in a longitudinal community-based pregnancy cohort, the All Our Babies study in Calgary, Alberta, completed open-ended survey questions providing explanations for the vaccination status of their child by 24 months postpartum. Qualitative responses were linked to administrative vaccination records to examine survey responses and recorded child vaccination status.

**Results:**

There were 1560 open-ended responses available; 89% (*n* = 1391) provided explanations for vaccinating their children, 5% (*n* = 79) provided explanations for not vaccinating/delaying, and 6% (*n* = 90) provided explanations for both. Themes were similar for those vaccinating and not vaccinating/delaying; however, interpretations were different. Two broad themes were identified: Sources of influence and Deliberative Processes. *Sources of influence* on decision making included personal, family, and external experiences. *Deliberative Processes* included risk, research, effectiveness, and balancing risks/benefits. Under *Deliberative Processes*, responsibility was a category for those vaccinating; while choice, instrumental/practical, and health issues were categories for those not vaccinating/delaying. Mothers’ levels of conviction and motivation provided a *Context* for understanding their decision making perspectives.

**Conclusions:**

Vaccination decision making is complex and impacted by many factors that are similar but contribute to different decisions depending on mothers’ perspectives. The results of this study indicate the need to examine new intervention approaches to increase uptake that recognize and address feelings of pressure and parental commitment to choice.

## Introduction

Vaccination contributes to significant reductions in communicable disease [1]. However, declining vaccination rates may be due to parental concerns resulting in increases in preventable disease [2]. Vaccination decision making is not simply a matter of two opposing viewpoints, but includes a spectrum from complete refusal to confident acceptance [3]. Parental vaccination decision making involves cognitive, psychosocial, and political factors influenced by current scientific, cultural, and media environments [4]. There continues to be a knowledge gap in how to best increase vaccination rates.

Currently, recommended childhood vaccinations in Alberta, Canada are not mandatory for school entry [5]. Parents may choose to vaccinate their child or not as they prefer. Vaccinations are publicly-funded requiring no personal payment nor health insurance. Public health nurses provide vaccinations in community-based clinics throughout the province, for example, the City of Calgary has 8 clinics covering all four quadrants of the city with 12 additional clinics in the communities surrounding Calgary [6]. Clinic hours cover week days and some evenings. Therefore, direct costs are not a barrier in Alberta to childhood vaccination. An understanding of the complex factors contributing to parental decision making is critical for designing and informing interventions to increase vaccination uptake.

The research question that guided this study was how do mothers explain their decisions to vaccinate, not vaccinate, or not fully vaccinate their children with the objective of understanding maternal vaccination decision making for infants up to 24 months.

## Methods

Survey data was obtained from the All Our Babies study (AOB), an ongoing longitudinal pregnancy cohort study in Calgary, Alberta [7]. Women were recruited in the second trimester of pregnancy when they provided written informed consent and completed mailed questionnaires during pregnancy and at 4, 12, 24, and 36 months postpartum. The AOB 24 month questionnaire, which was used for analysis, included both closed and open-ended questions on a variety of health topics, including childhood vaccinations.

We analysed mothers’ narrative responses to two questions related to routine childhood vaccinations. The first question asked “if your child has NOT received any or all of the vaccinations listed above, for what reason(s) did you NOT immunize your child?” The second question asked “if your child has received any or all of the vaccinations listed above, for what reason(s) did you immunize your child?” Qualitative inductive content analysis was used to develop codes, categories, and themes from the text responses [8]. We chose to bracket any theoretical frameworks or concepts related to parental immunization decision making during the analysis phase to mitigate preconceived notions and privilege participant voices and perspectives [9]. Initial codes were developed by one member of the research team (MM) guided by DM and further developed with two other investigators (VS, SM). These were then shared with the remaining team for verification. Over the course of several meetings of the full research team (*n* = 7) all members contributed to re-coding and theme development. Using an Excel spreadsheet, comparisons across sub-themes and themes were made to identify similarities and differences. Analysis continued until no new themes emerged. The research team consists of scientists, registered nurses (DAM, SM) and a physician (JDK) with a background in public health and immunization. DM and ST have experience in conducting qualitative studies including content analysis and phenomenology. The research team are interested in better understanding how immunization rates can be improved.

In analysing the data we were struck by the narrative of some parents that seemed to indicate a strong conviction and in others a limited conviction in their responses. As well, we also noted what seemed to be responses that indicated intrinsic or extrinsic motivation. Thus we quantified this data by developing definitions for conviction and motivation and then two team members (MM and DM) independently categorized responses and reached consensus for differences in ratings for both conviction and motivation. This analysis was done to provide context and background for the more inductive qualitative analysis.

Analysis was completed based on response to the questions posed in the survey to maintain integrity between questions and responses and to avoid oversimplified categorizations of parents. To verify vaccination status, the AOB survey data was linked to Calgary Zone Public Health administrative databases using unique public health numbers for those who completed the survey and agreed to linkage. However, a large proportion (*n* = 218, 14%) was not able to be linked. Ethics approval was obtained from the University of Calgary Conjoint Research Ethics Board (ID: REB14–0925).

## Results

### Participants

**Fig. 1:**
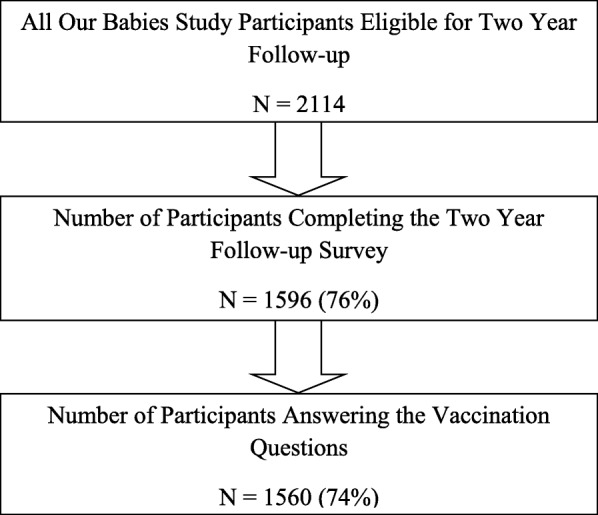
Participant flow chart

Narrative responses were available from 1560 (74%) of 2114 women eligible for the two year follow up survey (see Fig. 1). See Table 1 for participants’ socio-demographics at two years post-delivery. Of the 1560 women who responded, 1391(89%) provided explanations for vaccinating their children, 79(5%) for not vaccinating/delaying, and 90(6%) for both questions. We linked 1342 (86%) responses with administrative data to identify vaccination status; we were unable to link 218(24%) due to missing public health numbers. The majority (73%) of children completed all vaccinations by 24 months, 12% received some, and 1% received no vaccines (Table 2). Of the 79 women who explained why they did not or delayed vaccinating their children, 22% were partially or completely vaccinated.

**Table 1:** Descriptive information of the participants who completed the qualitative responses in the two year follow up survey (*n* = 1560)

Characteristics	n	(%)
Age		
24 or younger	34	[2]
25 to 34 years of age	900	(58)
35 or older	614	(39)
Missing	12	(< 1)
Marital Status		
Single	26	[2]
Married/Common Law	1503	(96)
Divorced/Separated/Widowed	31	[2]
Missing	0	(0)
Education		
Graduated High School or less	128	[8]
Complete or incomplete college, university, or trade	1176	(75)
Complete or incomplete post graduate studies	246	[16]
Missing	10	(< 1)
Born in Canada		
Yes	1249	(81)
No	302	[19]
Missing	9	(< 1)
Ethnicity		
White/Caucasian	1278	(82)
Chinese	58	[4]
South Asian	41	[3]
Filipino	30	[2]
Latin American	31	[2]
Black/African North America	22	[1]
Other	90	[6]
Missing	10	(< 1)
Total Household Income		
Less than $ 40,000	86	[6]
$ 40,000 - $ 60,000	110	[7]
$ 60,000 - $80,000	207	[13]
$ 80,000 - $100,000	226	[15]
$100,000 or more	657	(42)
Missing	274	[18]
Number of Children		
1	687	(44)
2	57	[4]
3 or more	22	[1]
Missing	794	(51)

**Table 2:** Vaccination status of child, according to vaccination registry, by mother’s response to the question of why vaccinated, why didn’t vaccinate, or if responded to both questions

	Response to Vaccination Questionnaire			
	Why n (%)	Why Not n (%)	Both n (%)	Total n (%)
Vaccination Registry Status				
None	1 (0.07)	21 (26.6)	0 (0)	22 (1)
Partial	108 (7.76)	17 (21.5)	62 (68.9)	187 (12)
Complete	1125 (80.9)	1 (1.27)	7 (7.8)	1133 (73)
Missing	157 (11.3)	40 (50.6)	21 (23.3)	218 (14)
Total	1391(100)	79 (100)	90 (100)	1560 (100)

Partial = received some Complete = received all by 24 months

### Themes

**Fig. 2:**
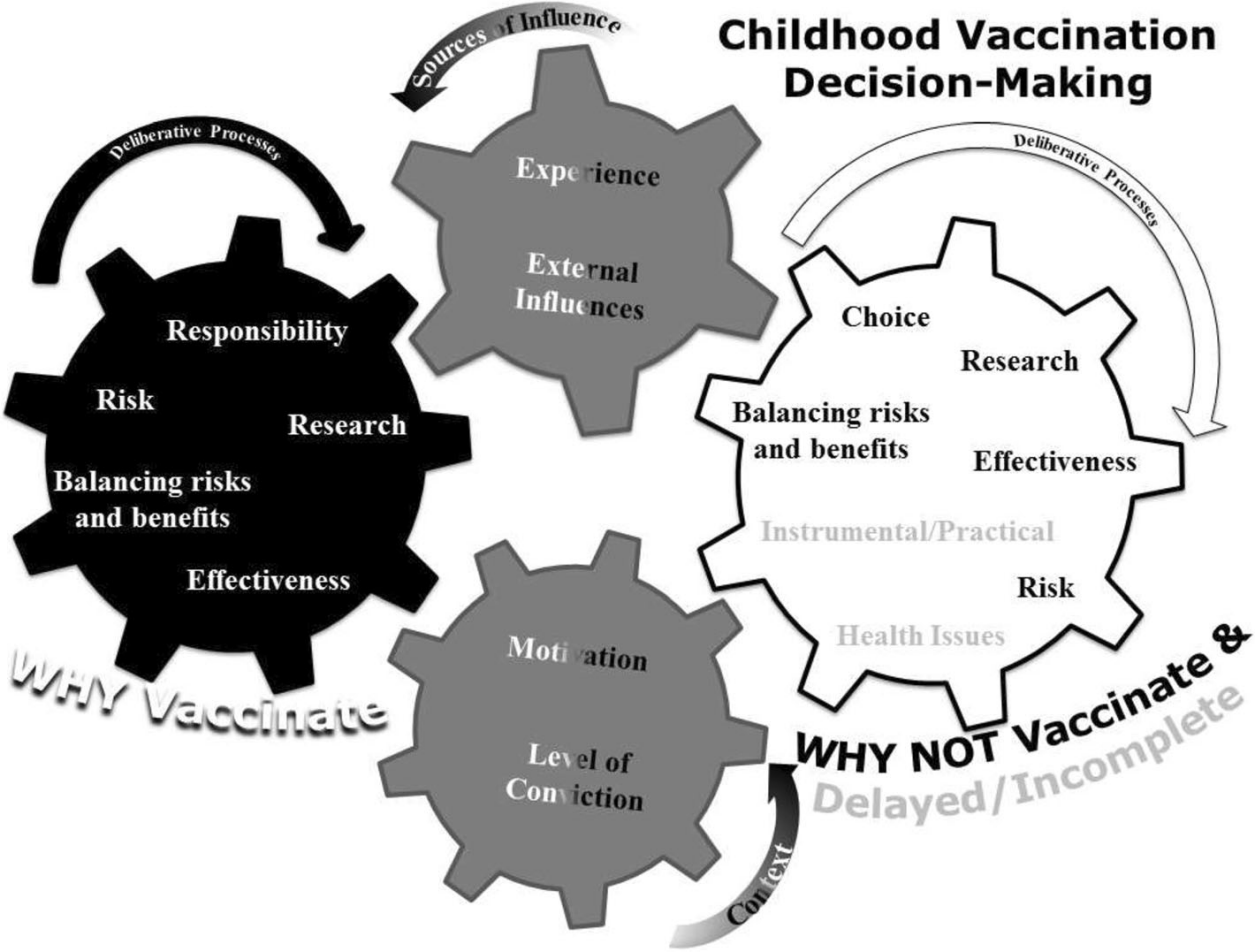
Visual model of themes extracted from participant responses

Two broad themes arose from sub-themes and categories (Fig. 2 and Table 3). The first theme, Sources of influence were those factors with potential to sway decision making. The second theme Deliberative Processes describes reasoning that went into the decision. We also identified that many parent responses reflected a high level of conviction and in others a high level of internal motivation for which we created operational definitions (see Table 4) and then categorized. These categorizations provide background context for understanding responses focused on the respondents’ internal deliberative processes. Level of conviction was reflected in certainty or uncertainty in response(s) and motivation conveyed how much decisions were internally or externally driven. For exemplars of themes see Tables 5 and 6.

**Table 3:** Themes derived from participant responses

Perspective	Why Vaccinate	Why Not Vaccinate/Delay
Theme 1	Sources of Influence	
Subtheme	*Experience*	*Experience*
Category	Personal and Others	Personal and Others
	Family member perspective on vaccinating	Family member perspective on vaccinating
Subtheme	*External Sources*	*External Sources*
Categories	Trust	Lack of Trust
	Pressured	Pressured
	Opportunity	Consulted with health professional
	Social norm	
Theme 2	Deliberative Processes	
Subtheme	*Risk*	*Risk*
Categories	Necessary	Unnecessary
		Natural immunity
		Immune system needs to mature
	Protection/Prevention/Precaution	
	Travel	
	Healthier with them	Healthier without them
	Safety	Safety
	Peace of mind	Concern about vaccine contents
		Number of shots
		Autism/ Allergy Issues
Subtheme	*Research*	*Research*
Category	It’s smart	
Subtheme	*Effectiveness*	*Effectiveness*
Category	Effective	Concern with effectiveness
Subtheme	*Balancing risks and benefits*	*Balancing risks and benefits*
Category	Benefits outweigh the risks	Risks outweigh the benefits
Subtheme	*Responsibility*	*Choice*
Categories	Personal	Personal
	Social altruism	
		*Instrumental/Practical*
		Just haven’t yet
		Difficult to take time off of work
		Busy clinic
		*Health Issues*
		Sick at time of shot
		Waiting until breastfeeding is complete
		Waiting for child to put more weight on

**Table 4:** Categories of parent conviction and motivation in statement about vaccination

Level	Why Vaccinate n (%)	Exemplars Why Vaccinate	Why Not Vaccinate n (%)	Exemplars Why Not Vaccinate
Level of Conviction				
Conviction	629 (42%)	“Felt it was the responsible medical decision given the potential health risks associated with the alternative” (810445)	91 (54%)	“We are doing homeopathic vaccines with naturopath” (710272)
Lack of Conviction	68 (5%)	“I was scared not to immunize but was not completely comfortable with it” (818019)	15 (9%)	“Did a lot of research and am somewhat worried about all the side effects.” (812348)
Unknown	784 (53%)	“immunity protection” (810450)	63 (37%)	“His father was against it, I am considering getting him immunized” (830900)
Motivation				
Intrinsic	248 (17%)	“in my opinion vaccinations are providing additional tools for the health of my child” (810524)	8 (5%)	“Choosing to delay vaccinations” (812381)
Extrinsic	268 (18%)	“Doctor’s recommendation” (812319)	104 (61%)	“Recommendation by naturopath to delay until 2 years if possible unless travelling. He stays at home and is not exposed to sick kids.” (810512)
Unknown	965 (65%)	“Good for us” (515024)	57 (34%)	“Husband is against vaccines and I am still ‘on the fence’” (818124)

Footnote. The definitions for types of motivation and conviction are as follows. Intrinsic motivation: decision to vaccinate/not vaccinate with no mention of an outside influence. Extrinsic motivation: indicating influence from another person aside from the husband, e.g. a doctor, naturopath, or extended family. Conviction: words or phrases that were clear either vaccinating, not getting their child vaccinated, not getting certain vaccines, or delaying all or some vaccines. Lack of conviction: contained words or phrases that showed uncertainty. Unknown: either too few words to tell or if statements were very general

**Table 5:** Why Vaccinate Themes and Exemplars

Theme	Sub-theme	Number of respondents affirming sub-theme Why Vaccinate	Representative Exemplars Why Vaccinate
Sources of Influence			
	Experience	45	“Something we’ve been doing with all our kids” (812467)
			“I was vaccinated as a child and I’m fine” (818606)
			“I have witnessed children contracting preventable diseases because of not getting vaccinations” (830893)
			“Grandfather had polio, we see the effects of this and chose to immunize” (810506)
	External Sources	86	“I had a Nazi doctor that told me do to it” (818454)
			“Mostly because of pressure from doctors and health nurses” (818194)
			“I trust that immunizations are important” (830404)
			“Expected by health community” (818362)
			“It is a general protocol in this country” (810647)
			“I believe it is a privilege to vaccinate your child and I am going to take advantage!” (818806)
			“Common sense” (515035)
Deliberative Processes			
	Risk	1234	“We felt the risks of not vaccinating her outweighed the risks of vaccinating her” (730265)
			“I felt it was necessary in this day and age” (818753)
			“Want the protection they provide” (818415)
			“Its what you do to protect your child” (818706)
			“If he wants to travel someday he’ll need all his shots so why not do it now” (830755)
			“Hard decision but figured with the amount we travel we should” (810612)
			“Overall health now and for the future” (818568)
			“For the best health of my child” (530213)
			“It’s what I believe the safest option” (818704)
			“Because I want to keep my child safe from a life threatening disease…” (810467)
			“I think it will give me peace of mind” (812590)
	Research	10	“Preventitive, did research-probably best thing to do especially with measles etc. on the rise. Also, some people depend on herd immunity” (815139)
			“Following recommended health guidelines. Plus, I researched it & agree with doing this” (530342)
			“Because its the smart thing to do.” (830464)
			“Because it’s smart. Science inconclusive about autism. Did much research.” (818582)
	Effectiveness	20	“Believe in science. Incredible amounts of research done tn ensure safety & effectiveness of vaccines. I think its socially irresponsible to not vaccinate children, leaves most vulnerable populations at risk for preventable disease” (812432)
			“Because it’s proven effective” (818633)
			“These illnesses are now rare due to the fact that we now have vaccinations” (810419)
			“Vaccines is why these diseases are under control” (815096)
	Balancing risks and benefits	35	“I felt the risk associated with not immunizing were greater than those associated with immunizing” (710295)
			“I felt the potential dangers of the vaccine were less than the potential dangers of the disease” (812369)
			“The likely hood of complicationss from vaccination is FAR outweighted by the benefits of vaccination” (510262)
			“I believe the benefits outweigh the risks and I don’t buy into the alarm over not vaccinating” (830445)
	Responsibility	233	“Public health” (660108)
			“It’s the right thing to do, just cause the disease is control doesn’t mean its safe not, vaccines is why these diseases are under control” (815096)
			“We think it is in the best interest of children & society as a whole to immunize against things we can” (830422)
			“For the better of all – common sense” (812551)
			“It’s our responsibility to society to prevent spread of disease” (818411)
			“To keep her from contracting any of the diseases and be a responsible member of society” (818661)
			“Protect against potentially fatal diseases. Also our public health duty” (830995)
			“I am a responsible parent” (818198)
			“The vaccine companies are heavily protected by gov’t” (812324)
			“Met with my doctor and a pediatric infectious disease specialist” (818020)
			“Recommendation by naturopath” (810512)
			“because it is the RESPONSIBLE thing to do” (818729)

**Table 6:** Why Not Vaccinate Themes and Exemplars

Theme	Sub-theme	Number of respondents affirming sub-theme	Representative Exemplars
Sources of Influences			
	Experience	17	“Also family members have had severe sideaffects to immunizations” (818539)
			“Everybody I have known has survived chicken pox w/o issues” (818086)
			“I have a background of Naturopath and herbs” (710232)
			“Know children who had terrible reactions” (812324)
	External Sources	8	“Felt pressured to do first 3 imm. But not anymore” (830369)
			“Not sure who to believe about pros and cons of vaccination” (818004)
Deliberative Processes			
	Risk	124	“There are many unknown dangers of getting it” (830847)
			“Vaccinations linked to health issues” (830753)
			“Do not agree with rigorous schedule at such a young age” (818688)
			“We are delaying until the immune system is better equipped at 7 yrs” (812405)
			“In our opinion they are not necessary and we do not want to expose our children to unnecessary foreign bodies” (810546)
			“Scared of autism gonna wait a few months” (818005)
	Research	12	“I have done my own research” (818020)
			“Concern with affect of intramuscular aluminum toxicity not being research enough” (830800)
			“I feel there’s not enough research on them” (830976)
	Effectiveness	3	“I don’t believe they are healthy or effective” (830402)
	Balancing risks and benefits	8	“Don’t believe they do more good than harm” (830680)
			“Cons outweigh the pros for vaccinating” (515051)
			“Am leaning towards not vaccinating. I think there is more harm than proven good” (812230)
	Choice	14	“Delaying by choice” (818638)
			“personal choice for both of us” (810555)
			“chose not to vaccinate” (818280)
	Instrumental/Practical	17	“My husband and I are working and not had the time yet” (812456)
			“He was in India for a few months, their kids have a different schedule for immunizations” (810563)
			“Busy clinic” (730238)
	Health Issues	18	“Born with numerous health issues, did not want to add to his problems” (812580)
			“Waiting for him to put more weight on” (810509)
			“Premature” (818057)
			“Rescheduled twice due to cough/flu” (830894)

#### Sources of influence

Vaccination decision making was influenced by personal, family or others’ *experiences,* i.e. generally a recall of reactions to vaccination or others’ perspectives on vaccinating, and *external sources*, such as health professionals or institutions. However, conclusions from those experiences and external sources varied depending on the type of experience and vaccination stance. For example, personal/family experience of those describing their decision to not vaccinate/delay typically referred to an experience that the mother or father had, as exemplified by a participant: “cause my brother died due to a reaction of vaccine and my dad in-law is alargic [sic] to eggs.” Another participant said “My mother very anti-vaccine- hard to get an appt. w/o her knowing.” On the contrary, personal and family experiences of those describing their decision to vaccinate was interpreted positively as seen in the following statements by two different mothers: “because both myself and oldest son have had them.” “My mum is a nurse and she’s big on vaccinations. I’m not 100% sure, but we did it to keep them safe.”

Some mothers experienced a feeling of pressure attributed to external sources. For example, those who chose not to vaccinate/delay were influenced by a doctor, nurse, naturopath, or pediatric infectious disease specialist with whom they discussed vaccinations. One respondent indicated their decision resulted from a “Recommendation by naturopath to delay until 2 years if possible unless travelling. He stays at home and is not exposed to sick kids.” Of note, another respondent said “The larger number of vaccines some of which are unnecessary (according to every medical doctor I’ve spoken to), the manner in which they are bundled and the fact that some contain monkey RNA and other foreign chemicals concerns me greatly in terms of my children’s immune systems.” Some participants experienced pressure to vaccinate their children, which resulted in discontinuing as the following exemplar indicates: “Decided not to continue. Felt pressured to do first 3 imm. but not anymore…!” In contrast, a participant whose decision was to vaccinate stated, “I was skeptical about immunizations but in the end decided to do it, mostly because of pressure from doctors and health nurses.” Many participants, who vaccinated, thought it was a requirement for either pre-school, day home, or the health authority.

Trust, a category of external Sources of influence for both decision making choices, varied by perspective. Lack of trust was evident in the following exemplar: there was a “…lack of ingredient and reaction reports released to the public. The vaccine companies are heavily protected by gov’t.” In contrast, another mother, indicated trust in the system, stating “Because I trust Canada Health and the World Health Org.”

For those who chose to vaccinate their children, additional external influencing categories included social norms and opportunity. Vaccinating was considered routine, regular, common sense, or standard by “following the norm of society.” Along the same lines, a category of opportunity emerged in which participants reported that vaccinations were available and “Immunizes [sic] are important and we are lucky to have them provided to us in this country.”

#### Deliberative processes

Mothers’ contemplated many factors and had to balance positive and negative perceptions. The perception of *risks* of vaccines and health and illness consequences of vaccinating or not were considered no matter the vaccination stance but conclusions drawn contrasted. For those who chose not to vaccinate/delay, there was a belief that vaccines were “unnecessary”; the child would be “healthier without them”; and that their children’s “immune system needs a chance to mature” and “get natural immunity.” In contrast, those who chose to vaccinate, felt it was “necessary,” as one respondent stated “to keep my child healthy.” Further, as one parent stated, “immunizations are important for children’s immune systems.” Travel was a factor for those stating why they vaccinated as one mother noted “when living in a multicultural society with so much travel it only makes sense.”

There were also contrasting views on the safety of vaccines. Some opposed to vaccination were “not comfortable with ingredients used in vaccines,” as one mother stated, “They’re toxic. I love my child.” Another concern regarding safety was the number of shots required as noted by one mother who stated “we don’t want to overload her system with too many vaccines.” In contrast, those in support of vaccines perceived them to be safe as one mother said “I think it will give me peace of mind,” another mother stated “…it’s better to have a safety net.”

Statements indicated *research* was done by parents to inform decision-making; however, each perspective lead to different conclusions based on how parents interpreted their own research or others’ research. Those opposing vaccines had a “Fear of possible link to autism/other diseases”; “we have done lots of research and vaccinating is not for our family” or made comments such as “While there isn’t conclusive proof that vaccinations are harmful, there are too many correlations to allergies and diseases for me to be comfortable.” Those in favor of vaccinating stated “Because it’s smart. Science… inconclusive about autism… Did much research.”

Those opposing vaccination had concerns with vaccine *effectiveness,* as one respondent indicated, “I feel there’s not enough research on them.” Those in favor identified vaccinations as “…an effective way of preventing disease” as well as reasoned that “…these illness are now rare due to the fact that we now have vaccinations.”

*Balancing the benefits and the risks* was a sub-theme evident in both perspectives. Those opposed to vaccination considered the likelihood of contracting diseases to be low and there was potential for more harm than good; their explanation was that “the risks outweigh the benefits” as one mother stated. Those in favor of vaccination were convinced that there was the potential for more good than harm, as one mom stated “because we value health and wellness, risk of not vaccinating is not an option in our home.”

The sub-theme of *responsibility* was only observed among those who were strongly in favor of vaccinations and either felt a “parental responsibility” or a “public health duty” and to “be a responsible member of society.” They also felt they “potentially contribute to eradication of disease.”

The sub-theme of *choice* was only observed among those indicating no/delayed vaccination. A mother stated that “I chose not to have the live vaccine at this time.” Often the personal choice comments did not indicate an explanation merely stating that it was “…MY CHOICE.”

The sub-theme of *instrumental or practical* reasons was evidenced by a number of parents indicating that their child’s vaccination was delayed/incomplete and made comments such as “will get them done, just haven’t yet”, with no other indication that vaccinating was unacceptable. While others identified practical issues like “too hard to fit in when working.” Another practical issue was clinics being very busy and “backed up” leading to delay. The sub-theme of *health issues* arose from mothers who indicated they would vaccinate when “no longer breast feeding” or when their child “put on more weight.”

#### Context

A mother’s *level of conviction* and *motivation* was considered as context in which explanations were provided (see Table 4). Approximately 40% of participant responses conveyed a sense of either intrinsic or extrinsic motivation regardless of their decision. There were a greater proportion of ‘Why Not vaccinate’ respondents who we interpreted as being externally motivated (61%), consistent with theories of social network influences on those who are vaccine hesitant [18]. For those who chose to delay/not vaccinate, statements such as “We wanted to wait until she was older to do her vaccinations” and “Because I did not want to” demonstrate intrinsic motivation. While statements such as “Recommendation by naturopath to delay…” and “We are delaying immunizations – our family (extended) is completely against them” show how external sources influenced motivation not to vaccinate. There were also examples of both internal and external motivation for those who responded ‘why vaccinate’, for example “I didn’t really think twice about it. I’d already made up my mind to do it” contrasting with “…as suggested by community health.”

For some mothers (~ 50%), a strong level of conviction was evidenced regardless of vaccination decision while the remainder of the sample responses lacked evidence of strong conviction. There were a greater proportion of ‘Why Not vaccinate’ respondents who we interpreted as having strong conviction (54%). One mother who indicated a strong conviction against vaccinating stated “Decided not to continue…It’s MY CHOICE.” Others indicated ambivalence, “Not sure who to believe…” and “He has had them all but I am not a believer that it is a good thing.” A mother in support of vaccinating who showed conviction stated “I strongly believe in it…” while ambiguity can be observed when a mother said “because it’s a good idea, even though I don’t like it.”

## Discussion

Our analysis and results provide a detailed description that contributes to an understanding of maternal vaccination decision making and can provide guidance to decision makers on approaches to take when designing interventions to increase vaccination uptake. Our study highlights the dynamic nature and multilayered factors that contribute to parental decision making [10, 11]. Across both major themes (Sources of Influence and Deliberative Processes), many sub-themes were the same regardless of the mother’s vaccination decision; however, interpretation was different. A few sub-themes (*responsibility, choice, instrumental/practical, and health issues*) were unique to the particular decision making perspective. We did not explicitly set out to study vaccine hesitancy. However, our findings include certain elements found in existing hesitancy frameworks. For instance, the range of parental responses are similar to those described by Leask as spanning from refuser to confident acceptor [12]. As Gowda and Dempsey identify, even those parents whose children are fully immunized have concerns, and these were evident in our results [13].

Those who had timely completion of vaccination for their children were positively influenced by their personal experiences or experiences of their family, similar to other studies [14–17]. These explanations are consistent with existing child care decision making frameworks [10], as well as vaccine decision making frameworks [18, 19]; in which social networks are seen as sources of influence in decision making and thought to be an avenue for interventions. Basing decisions on personal experience is also consistent with psychological decision making frameworks contending that perceptions and judgements, particularly in situations of uncertainty, are more likely influenced by information that is familiar, salient and recent [10]. Information from health care providers created the impetus, and in some, the feeling of pressure to complete vaccinations. The idea of conformity [20] or feeling pressure [21] has been previously described. Similar to findings by Tickner et al. and Wilson et al., vaccination was considered part of the social norm or was thought to be a requirement for day care or school attendance, although this is not true in Alberta [16, 17]. We identified vaccination contributed to peace of mind, which is a new explanation for vaccinating as far as we are aware. Similar to the findings of Tickner et al. and others [12, 16, 19], it was not only a personal but also societal responsibility.

The reasons, for those responding to ‘Why Not vaccinate’, included negative experience with vaccination or lack of impact of vaccination as a reason for their decision; a finding consistent with past research [14, 16, 17, 21, 22]. Similar to previous studies, we found that when parents received information from health care professionals that supported their own negative views or pressure they experienced to vaccinate, this contributed to them deciding to not/delay vaccinating their children [17, 20]. This highlights the phenomenon of parents choosing health care providers who have consistent views with their own [23]. Busse et al. found parents wanted unbiased or neutral information from physicians [21]. This finding reinforces the need to address health care provider doubts and knowledge gaps because of the influence they wield [21].

Some parents in our study perceived that risks outweighed benefits and that their children would be healthier without being vaccinated [14, 17, 24, 25]. Others expressed concern regarding the ingredients of vaccines and a fear of autism, which has been cited in a number of previous studies [14, 16, 22, 24, 26, 27]. Prospect theory provides an understanding indicating that fear of loss or harm creates more intense responses; and supports our results regarding level of conviction, with more parents responding to Why Not vaccinate with conviction [10]. We identified a lack of trust in health systems/the government and organizations; a common theme in other research [14, 16, 20, 24–26, 28, 29]. In contrast, those who responded ‘why they vaccinated’ expressed trust in the government, the system, and their health care provider. Vaccine effectiveness was questioned by parents, as has been previously identified [17, 22]. It is important to note that personal choice was only used as a reason for those responding Why Not Vaccinate and indicates that pressure tactic types of vaccination campaigns can have an opposite impact for some parents. Other research has not focused on personal choice as a key theme but on religious [14, 25, 29] or ethical beliefs [24] influencing decision making. Practical considerations were cited by parents who had delayed, such as difficulty taking time off work for appointments, similar to two previous studies [16, 29]. For those parents who indicated they were delayed without providing any rationale, it is unclear why they did not prioritize vaccination. It was unclear whether this pertained to their perspective of vaccination i.e. acceptance, or if the reasons were more practical in nature.

Analogous to our study, previous qualitative and quantitative studies on parental perspectives included parents who had fully immunized as well as parents whose children were partially or not immunized [14, 15, 17, 20, 21, 25, 27]. Parents from each perspective reported doing research on their own, as Tickner et al. found, for those who chose to vaccinate and others have found for those who chose not to vaccinate [16, 22, 24, 26, 28–30]. Contrary to other studies, parents responding both Why and Why Not vaccinate in our study did not describe needing more information; [16, 31] they researched on their own but a few in each group identified the need for more research/studies to be conducted. The use of the internet for “research” to support decision making is concerning as non-medical websites include inaccuracies, rumors, and myths that contribute to fear and hesitancy [16]. The participants described by Luthy et al. as being anxious about vaccination may be similar to our participants who responded to both questions in the survey i.e. describing why they vaccinated and why they did not [24].

### Strengths and limitations

This study’s strengths include a large sample for a qualitative analysis, although our findings may not differ from others they confirm those findings in a large and robust data set.. Close to 75% of the eligible study population responded and 98% of those who completed the survey answered the vaccination questions. The sample demographics were consistent with those of the urban center from which the sample was recruited, and the data were collected concurrently with the vaccination timeframe, reducing the potential for recall bias. Multiple investigators read the participant responses and corroborated the themes and sub-themes in an iterative fashion for accuracy of interpretation. Novel to this study, the survey questions asked about both perspectives of vaccination allowing for the analysis to include explanations for both types of decisions, and enabling participants to communicate if they did not feel strongly about just one perspective. Other studies [15] classify parents into categories based on vaccination decision; conversely, this study did not label parents but instead created a visual representation of their feelings, opinions, and explanations for or against childhood vaccination in order not to set up opposing “sides” or create labels.

The results of this study reflect a homogenous sample of well educated, higher income mothers and thus may not be applicable to others with different demographics or contexts. Data were comprised of text responses to a survey thus there was no opportunity to probe responses to gain a more in-depth understanding of the vaccination perspectives. The quantification of motivation and level of conviction that provided context for the analysis was limited by the authors’ interpretations without being able to further explore or test the assumptions made in the categorizations and thus need to be interpreted with caution.

### Implications and future research

Based on our findings, confirming previously identified issues with vaccination, we propose two strategies to combat vaccine hesitancy. The first is to examine the usefulness of motivational interviewing. Health professionals are often the first point of contact for vaccinations and are integral to implementing strategies for increasing vaccination uptake [12]. Motivational interviewing could be used to address the feelings of pressure experienced by parents and the voiced commitment to parental choice that our results identify. Parents might feel more understood [20] and the conversation less likely to contribute to negative and lasting parent impressions.

The second strategy would be to explore use of storybooks to target parental attitudes towards childhood vaccination. This technique has been used with parental attitudes towards children’s oral health behavior and found to be effective [32]. The premise is that the story is written for children but subtly gets parents to think about their attitudes towards the subject. An example could be the use of a plot in which a character is unable to get vaccinations due to medical reasons and the story could show how others can vaccinate to protect that child. To our knowledge this has not yet been researched but could be of interest to study as an intervention.

## Conclusion

This qualitative analysis of contemporary survey data provided an understanding of urban parental vaccination decision making concurrently while parents were making those decisions. Vaccination decision making is complex and can be impacted by the interplay of Sources of Influence, Deliberative Processes, and context that are similar but interpreted differently depending on a parent’s vaccination stance. Using approaches to enhance paradigms of collective responsibility may influence those hesitant parents with social responsibility beliefs. Addressing instrumental issues through vaccine delivery systems or approaches such as text reminders may address barriers for those experiencing obstacles to access. Most importantly, recognising the careful contemplation of those who choose to delay/not vaccinate their children is important for health professionals to consider in conversations with parents about vaccinations. Future research could include testing the effectiveness of motivational interviewing and the use of story books to increase vaccination uptake.
